# DMS-YOLO: Small target detection algorithm based on YOLOv11

**DOI:** 10.1371/journal.pone.0341991

**Published:** 2026-01-30

**Authors:** Minyu Huang, Wengang Jiang

**Affiliations:** School of Automation, Jiangsu University of Science and Technology, Zhenjiang, Jiangsu, China; Northwestern Polytechnical University, CHINA

## Abstract

To address the challenges in vehicle detection from unmanned aerial vehicle (UAV) overhead images, such as small object size, low resolution, complex background, and scale variation, this paper proposes several targeted improvements to the YOLOv11n model. Firstly, inspired by the Cross Stage Partial Networks (CSPNet), a Dynamic Multi-Scale Edge Enhancement Network (DMS-EdgeNet) is designed to improve robustness to local target features. This module applies multi-scale pooling to extract edge features at various scales and dynamically fuses them through adaptive weighting. Secondly, the DynaScale Aggregation Network (DySAN) module is introduced into the neck network, and a multi-level jump connections structure is adopted to fuse low-level and high-level boundary semantics, thereby improving the detection capability of fuzzy boundary targets and improving target positioning accuracy under complex imaging conditions. Finally, a P2 small target layer is added to further improve the accuracy of small target detection. Based on these innovations, we propose a new architecture named Dynamic Multi-scale and Channel-scaled YOLO (DMS-YOLO), significantly improve the model’s ability to perceive small targets. Experimental results show that DMS-YOLO improves mAP50 and mAP50-95 by 7.0% and 2.9%, respectively, on the Aerial Traffic Images dataset, and by 5.1% and 3.1% on the VisDrone-DET2019 dataset, demonstrating superior performance over the YOLOv11n baseline.

## Introduction

With the continuous development of domestic drone technology and remote sensing imaging technology, the production cost of drones has been steadily decreasing. Target detection technology based on aerial imagery has been widely adopted and is extensively used in everyday life and engineering fields, including urban planning [1], hydrological monitoring [2], traffic control [3], and pedestrian detection [4]. However, there are still limitations when directly applying these techniques to small object detection: first, small objects in images have low resolution and blurred texture features, making it difficult for shallow networks to extract effective semantic information; second, small objects in aerial images often exhibit characteristics such as dense distribution, occlusion, or random arrangement directions, which can lead to missed detections or false positives; additionally, the low contrast between complex backgrounds (such as roads, building shadows, and vegetation coverage) and targets further increases the difficulty of feature separation. Therefore, designing a small object detection algorithm suitable for aerial imagery holds significant research value and importance.

Object detection is generally divided into traditional object detection and deep learning-based object detection methods. Traditional object detection algorithms include the Viola-Jones algorithm [5], DPM algorithm [6], and HOG+SVM algorithm [7]. Traditional object detection consists of three components: region selection, feature extraction, and classification. For example, region selection commonly uses the sliding window method, but since the size of the detection target is unknown, it is necessary to design windows of different sizes and proportions for sliding detection, and the step size must also be appropriately selected. Additionally, manually designed features exhibit poor robustness for multi-scale targets, resulting in low detection efficiency, poor scale adaptability, susceptibility to complex backgrounds, and reliance on large datasets. These drawbacks limit the application of traditional detection methods in aerial image recognition.

With the continuous advancement of deep learning technology, deep learning-based object detection has begun to be widely applied and has achieved significant results. Deep learning-based object detection algorithms automatically learn multi-level feature representations of images through convolutional neural networks (CNNs), making object detection in complex environments more robust. Currently, deep learning-based object detection is divided into two categories. The first category includes single-stage detectors such as YOLO [8] and SSD [9], which achieve improved detection speed through end-to-end architecture design. The other category includes two-stage detectors represented by Faster R-CNN [10], which first generate candidate boxes via a region proposal network (RPN) and then perform classification and regression on the candidate regions, offering advantages in terms of accuracy. Among these detection algorithms, YOLO stands out for its excellent detection speed and accuracy, as well as its lightweight parameter count.

However, YOLOv11 suffers from issues such as loss of small object features and imbalanced feature fusion when handling multi-scale object problems. In the context of drone aerial imagery, “multi-scale” refers to two related aspects: (1) object scale variability, where objects of the same category appear at significantly different sizes in images due to variations in drone altitude, viewing angle, and shooting distance; (2) feature layer multi-scale, where detection networks employ multiple feature layers at different resolutions (e.g., P2, P3, P4, P5 in YOLO), but small object detail information is easily lost during the fusion process between these different-scale feature layers, leading to inadequate feature representation for small objects. Recent studies improve cross-scale fusion through cross-layer connections and dynamic feature refinement [11]; nevertheless, semantic redundancy during fusion and limited long-range dependency modeling may still hinder robustness across diverse object sizes.

YOLOv11 also suffers from issues such as large localization errors for edge-blurred targets (e.g., motion-blurred scenes). Due to blurred edge features and lost detail information, the model struggles to accurately capture target contours, leading to localization deviations and reduced detection accuracy. Several works improve localization under blurred boundaries by optimizing IoU-based loss functions and introducing focusing mechanisms [12,13], yet robustness for extremely blurred targets remains challenging.

Small objects are commonly defined either by absolute pixel size or by their size relative to the whole image. For instance, the COCO dataset [14] defines objects smaller than 32×32 pixels as small objects, while the DOTA dataset [15] defines objects with pixel dimensions between 10 and 50 pixels as small objects; however, there is no universal standard across datasets and applications. In UAV aerial imagery, small targets typically occupy only a few pixels with weak texture cues, and their details are easily suppressed by downsampling and cross-scale fusion, resulting in severe feature loss and large localization errors, especially under complex backgrounds and blurred boundaries.

Existing studies mainly follow two directions. One direction enhances feature representation for small targets via feature enhancement or attention mechanisms [16,17]. The other direction mitigates information loss by improving multi-scale feature fusion or preserving high-resolution representations [18,19]. These approaches have been shown to improve small-object detection performance.

To address these challenges, this paper proposes the lightweight DMS-YOLO, with the following specific work:

1. To address the issue of varying target sizes when capturing images at different angles and heights, which increases detection uncertainty, this paper designs the lightweight C3K2 Dynamic Multi-Scale Edge Enhancement Network (C3K2-DMS-EdgeNet) module to address target scale changes and conducts position experiments to determine the optimal placement of this module within the model.
2. To address the issue of feature information loss caused by blurred edges of small objects, this paper designs the DynaScale Aggregation Network (DySAN), which dynamically adjusts the feature scale aggregation strategy to enhance the capture of texture details and edge information of small objects, promoting the effective fusion of low-level semantic features and high-level semantic features, and improving detection capabilities for small objects with low pixel density and blurred edges in complex backgrounds.
3. In the design of the Neck network, this paper abandons the traditional unidirectional feature pyramid structure and integrates the top-down approach of FPN with the bottom-up approach of PANet. A multi-level jump connections structure similar to BIFPN is proposed to achieve multiple, bidirectional circular flows of features between deep semantics and shallow details.
4. To further improve the accuracy of small object detection, this paper introduces a P2 small object layer in DMS-YOLO while maintaining a balance between detection efficiency and accuracy.

## Materials and methods

### Baseline model: YOLOv11n

**Fig 1:**
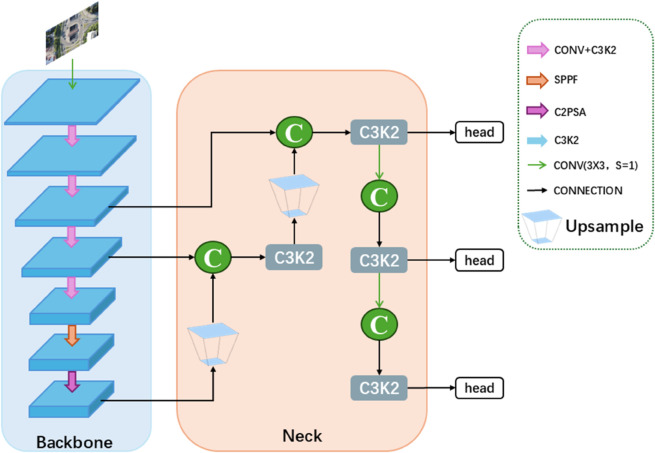
Structure of YOLOv11.

YOLOv11, the new object detection algorithm launched by Ultralytics on September 30, 2024, continues the “real-time and efficient” characteristics of the YOLO series. YOLOv11 offers five models of different sizes: n, s, m, l, and x, with the number of model parameters increasing sequentially and accuracy improving accordingly. The model primarily consists of Backbone, Neck, and Detect. By decomposing the network into independent functional modules, it achieves decoupling between modules, which are then assembled via configuration files to enable “plug-and-play” functionality. Additionally, YOLOv11 introduces two innovative modules compared to previous versions: the C3K2 module and the C2PSA module. The C3K2 module inherits from the C2f module in YOLOv8 and replaces it. The C3K2 module combines CSP to enhance gradient flow and improve feature extraction capabilities. Following the original SPPF layer, the C2PSA module is added, integrating a multi-head attention mechanism, and integrates a reusable stacked Pyramid Slicing Attention (PSA) module to enhance the capture of key features. Additionally, in the decoupled head’s classification branch, Depthwise Separable Convolution (DWConv) is used to replace standard convolution, reducing parameter count and computational complexity while maintaining classification accuracy. The YOLOv11 architecture is shown in Fig 1.

Among the five YOLOv11 model sizes, YOLOv11n is the lightest and fastest in terms of detection speed. Therefore, this paper selects YOLOv11n as the baseline model.

### Proposed method: DMS-YOLO

**Fig 2:**
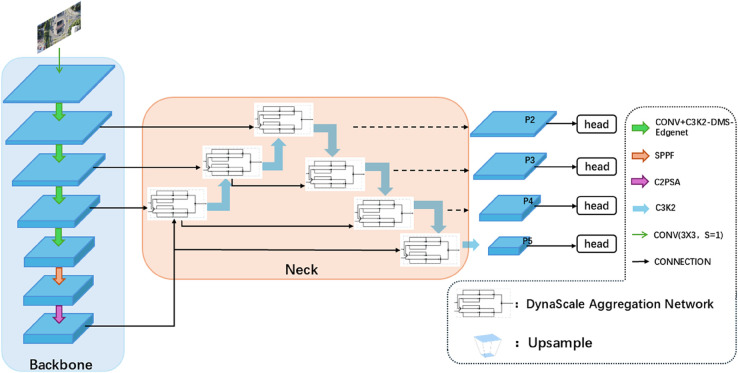
Structure of DMS-YOLO.

Based on the YOLOv11n baseline model, to address the issues of missed detections or false positives caused by the large variation in target distribution, diverse sizes, and unclear features during drone aerial photography, this paper proposes DMS-YOLO, an improved small object detection algorithm based on YOLOv11n, whose structure is shown in Fig 2. First, the C3k2 in the backbone network is improved by incorporating the CSP concept, the DMS-EdgeNet is designed to replace the Bottleneck in C3k2; Then, the DySAN module is introduced into the neck network to replace upsampling and channel concat; Finally, the P2 small object detection layer is added to the detection head to further enhance the algorithm’s ability to extract features of small objects.

#### C3K2-DMS-EdgeNet.

**Fig 3:**
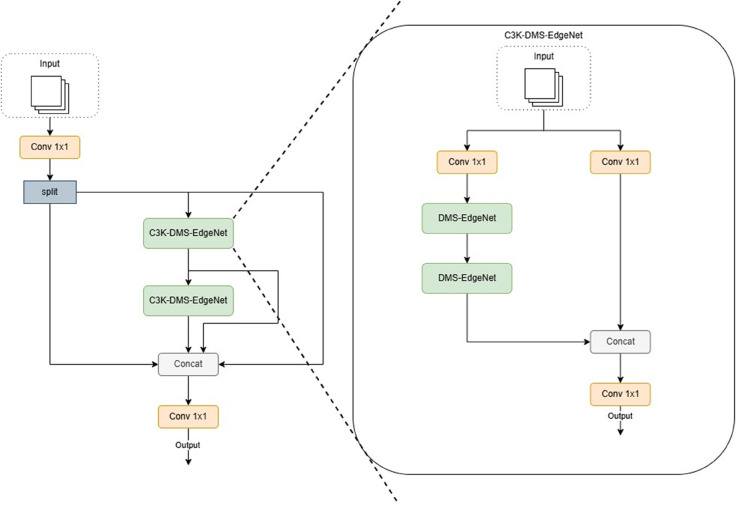
Structure of C3k2-DMS-EdgeNet.

As mentioned by Yangang Li et al. [20], drones can adjust their speed, direction, and altitude during aerial photography to capture images from various angles, such as bird’s-eye view, tilted view, and side view. This diversity often results in the same detection target appearing in different sizes and appearances in different images, thereby increasing detection uncertainty. Therefore, to address target scale changes, this paper replaces the Bottleneck module in the C3k2 module with DMS-EdgeNet, as shown in Fig 3.

**Fig 4:**
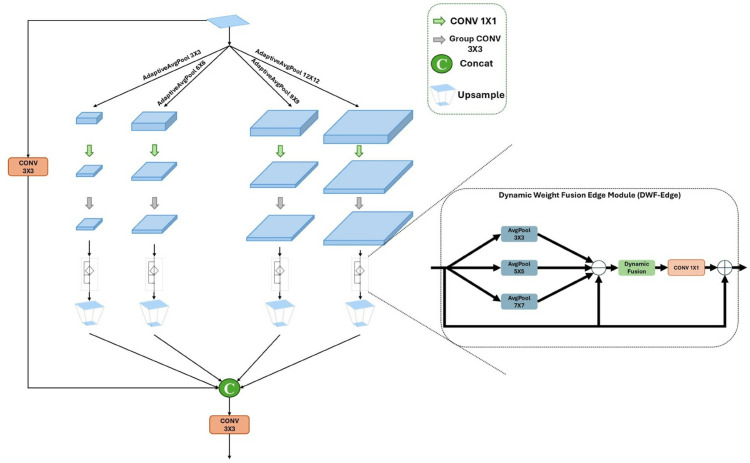
Structure of DMS-EdgeNet.

As shown in Fig 4, the DMS-EdgeNet architecture generates feature maps of different scales through adaptive average pooling of different sizes(3×3, 6×6, 9×9, 12×12). Each branch contains a DWF-Edge that enhances edge information at multiple scales. Combining the concept of dynamic fusion, the DWF-Edge module uses multi-scale pooling kernels (3×3, 5×5, 7×7) to extract edge features of different granularities and employs dynamic weight generation (convolution with Sigmoid activation) to adaptively fuse edge information across different scales. This enables feature-driven automatic adjustment of the contributions from the three scales (large, medium, and small), avoiding the need for manually setting fixed weights, thereby enhancing the flexibility in capturing edge features. This design ensures that small objects can be effectively detected at different scales. Finally, feature alignment is performed using upsampling, followed by fusion of features at different scales.

**Fig 5:**
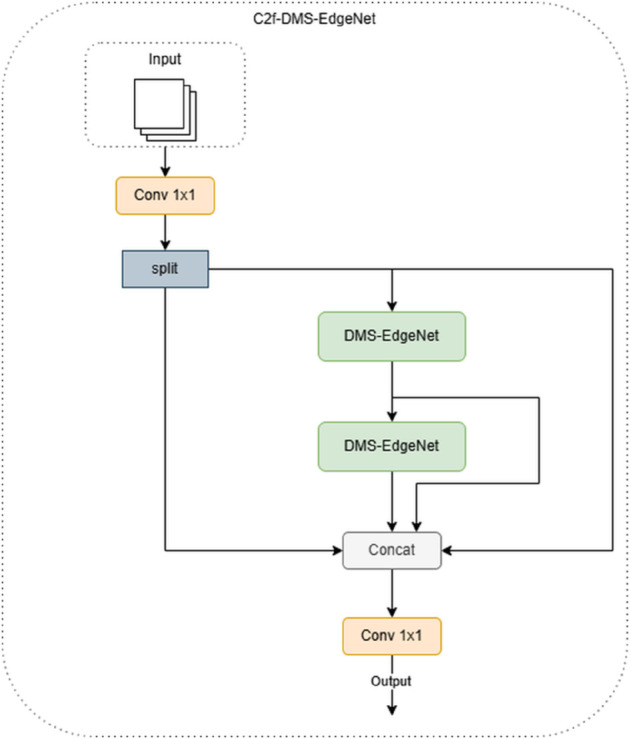
Structure of C2f-DMS-EdgeNet.

When designing the backbone network architecture, the C3k2 module requires manual selection to determine whether to use the more complex C3k structure. When the parameter C3k=False in C3k2 in the configuration file, the C3k2 module automatically degrades to a C2f module. In the backbone structure of the DMS-YOLO designed in this paper, the first two C3k2 modules do not use the C3k structure but directly adopt the C2f framework. The Bottleneck in C2f is directly replaced with DMS-EdgeNet. This is because the shallow layers of the backbone network need to process high-resolution inputs, and the C2f structure significantly reduces computational complexity by simplifying cross-stage connections and reducing redundant branches. Compared to using C3k, C2f is more suitable for early feature extraction and avoids computational bottlenecks caused by complex operations. Experimental results show that using C2f-DMS-EdgeNet in the shallow layers outperforms the C3k2 structure using C3k in terms of the performance metric mAP-50, while the number of parameters slightly decreases, validating the effectiveness of this design. C2f-DMS-EdgeNet is shown in Fig 5.

The dynamic fusion process in DWF-Edge is as follows:

The input feature map $X\in {\mathbb{R}}^{C\times H\times W}$ undergoes three average pooling operations at different scales to extract its low-frequency information. Then subtract the features obtained through average pooling from the original input features to obtain enhanced edge information:

$$E_{s}=X-{AvgPool}_{s}(X),s\in \{3,5,7\}$$ (1)

Where “${AvgPool}_{s}$” represents the size of the kernel as s×s, Padding is ⌊s/2⌋.

After extracting three edge information, adaptive weights are generated through the convolution layer and sigmoid activation function:

$$W=\sigma \left(Conv_{1\times 1}\left(Concat\left(E_{3},E_{5},E_{7}\right)\right)\right)$$ (2)

Subsequently, the generated adaptive weights are used to adaptively fuse multi-scale edge features:

$$E_{fused}=W⊙E_{3}+\left(1-W\right)⊙\frac{E_{5}+E_{7}}{2}$$ (3)

Where ⊙ denotes channel-wise multiplication. The adaptive weight W is directly involved in the process of feature weighting. Its gradient is propagated backward through backpropagation to the preceding 1×1 convolutional layer, driving weight parameter updates to dynamically adjust the contribution of edge information at different scales.

Finally, the fused features are convolved and enhanced, then added to the original input to obtain the final output:

$$Y=X+Conv_{3\times 3}(E_{fused})$$ (4)

#### DynaScale aggregation network.

**Fig 6:**
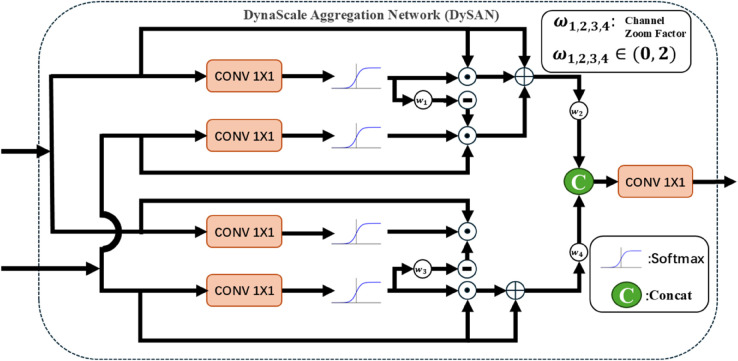
Structure of DySAN.

As mentioned by Hongxing Ping et al. [21], once a drone reaches a certain altitude, subsequent challenges arise, with the most significant challenge being the detection of targets that are very small in the image. Additionally, Shuang Zeng et al. [22] noted that when targets become very small, issues such as insufficient texture features, inadequate low-level semantic features, and missing high-level feature information arise. Furthermore, Kang Tong et al. [23] pointed out that small targets lack the necessary information to distinguish them from complex backgrounds, as small targets in high-altitude images have low pixel density and blurred edges. Therefore, to address the issues of blurred edges in small targets and insufficient integration of high and low-level semantic features, we propose the DySAN, whose structure is shown in Fig 6.

This module is designed by mimicking the biological process of distinguishing targets from backgrounds. Specifically, it combines deep and shallow features, refines object boundaries, and enhances boundary features, effectively addressing the issue of blurred target boundaries, as mentioned by Xiangtai Li et al. [24]. Shallow features have less semantic information but high resolution, low distortion, rich details, and clear boundaries. Deep features contain rich semantic information but have low resolution. Therefore, directly fusing the two may lead to redundancy and inconsistency. However, the DySAN module effectively addresses this issue. The DySAN module process is as follows: The input features $F_{1}\in {\mathbb{R}}^{C\times H\times W}$ and $F_{2}\in {\mathbb{R}}^{C\times H\times W}$ represent the shallow and deep-level features, respectively. Weights are generated through linear mapping and the Sigmoid function:

$$F_{1}^{\prime }=Conv_{1\times 1}\left(F_{1}\right),F_{2}^{\prime }=Conv_{1\times 1}\left(F_{2}\right)$$ (5)

where $Conv_{1\times 1}$ is convolutions with a convolution kernel of 1, used to reduce the number of channels to 32.

However, the weights generated by the Sigmoid function cannot fully capture the differences in feature importance across different input scenarios, resulting in overly conservative feature representation. This is particularly true for images captured by aerial drones, where the weight upper limit of 1 restricts the potential for enhancing the contribution of certain layers (e.g., shallow details of small objects). In deep semantic processing, when noise is present, the weight upper limit of 1 may prevent noise amplification, thereby failing to completely eliminate noise influence. To address these issues, four dynamic scaling factors $w_{1},w_{2},w_{3},w_{4}\in (0,2)$ are introduced to achieve path-specific fine-grained control. The DySAN process is detailed as follows:

As shown in Fig 6, the DySAN module is divided into two identical upper and lower units, each of which performs the following operations:

$$UNIT(F_{1},F_{2})=F_{1}^{\prime }⊙F_{1}+F_{2}^{\prime }⊙F_{2}⊙(⊝F_{1}^{\prime }\times w_{i}))+F_{1}$$ (6)

Where ⊙ denotes element-wise multiplication and i∈{1,3}, the final output of the DySAN module is:

$$Output=Conv_{3\times 3}\left(Concat\left(UNIT(F_{1},F_{2})\times w_{2},UNIT(F_{2},F_{1})\times w_{4}\right)\right)$$ (7)

Where *F*_1_ represents deep semantics and *F*_2_ represents shallow semantics, after concatenation, the output is obtained through convolution with a convolution kernel of 3.

**Fig 7:**
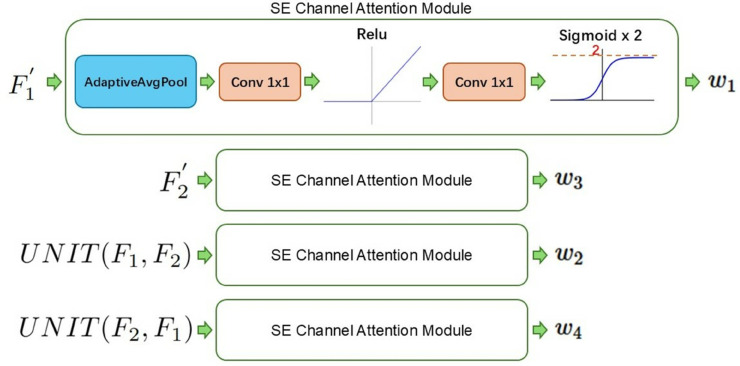
Dynamic scaling factor generation via SE attention.

For the four dynamic scaling factors, this paper employs the Squeeze-and-Excitation (SE) attention mechanism [25], a classical attention mechanism, to dynamically generate path-specific scaling weights. The process is shown in Fig 7.

In particular, to enable the network not only to suppress features but also to enhance critical features, we multiply the output of the Sigmoid activation by 2, expanding its range from (0,1) to (0,2). As a result, the four generated scaling factors ($w_{1},w_{2},w_{3},w_{4}$) possess content-adaptive capability and broader dynamic range adjustment capacity, thereby achieving refined control over different feature fusion pathways.

Finally, the neck network uses multi-level jump connections (P4→P3→P2→P3→P4→P5) to achieve top-down and bottom-up bidirectional enhancement. It refines high-level semantics to shallow layers (P4→P3→P2) to improve the model’s detection accuracy for small targets, and then feeds shallow layer details back to deep layers (P2→P3→P4→P5) to repair blurred boundaries. This multiple cross-layer interaction reduces information decay in a single fusion, facilitates continuous detection of small targets, and forms a closed-loop feature enhancement.

#### P2 Small target layer.

In images captured by drones, small targets typically account for the majority of the dataset due to the shooting angle and altitude [26]. Therefore, a new P2 small target layer was added. The high-resolution features of the P2 layer contain more high-frequency information such as edges and textures, retaining more boundary information of small targets, making it suitable for locating small targets. Experimental results show that introducing the P2 small target layer effectively enhances the model’s ability to handle small target detection tasks, reduces the probability of missed detections and false positives, while maintaining a balance in detection efficiency.

### Datasets

This paper utilizes two datasets. The first is the open-source dataset Aerial Traffic Images available on Kaggle (https://www.kaggle.com/datasets/cihangiryiit/aerial-traffic-images), created by the Roboflow team, which provides aerial traffic imagery captured by drones. The dataset contains a total of 2,708 images, including 1,710 training images, 558 validation images, and 440 test images, roughly divided into a 6:2:2 ratio. This dataset includes eight categories: PMT, buses, cars, trucks, vans, minibuses, motorcycles, and articulated buses. Specifically, it contains 67 PMT images, 6,715 bus images, 64,495 car images, 3,341 truck images, 3,270 van images, 1,687 minibus images, 3,154 motorcycle images, and 1,257 articulated-bus images. The dataset contains a large number of small objects with uneven data distribution and includes numerous low-light scenarios, making the detection task more challenging.

**Fig 8:**
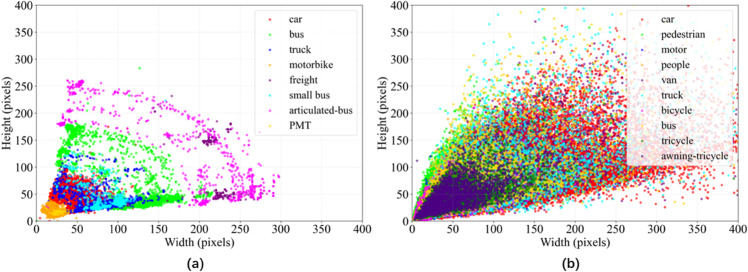
Target size distribution of the dataset. (a): Distribution of target sizes for the dataset Aerial Traffic Images. (b): Distribution of target sizes for the dataset VisDrone-DET2019.

The second dataset is VisDrone-DET2019 [27], created by the Machine Learning and Data Mining Laboratory at Tianjin University in China. It contains a total of 10,209 images, including 6,471 images in the training set, 548 images in the validation set, and 3,190 images in the test set, roughly divided into a 6:1:3 ratio. This dataset includes 10 categories: pedestrian, people, bicycle, car, van, truck, tricycle, awning-tricycle, bus, and motor, totaling 10 categories. Similar to the challenges faced by the Aerial Traffic Images dataset, the difficulties in VisDrone-DET2019 also lie in detecting small objects and dealing with uneven data distribution. The size distribution of objects in both datasets is shown in Fig 8(a) and 8(b).

### Experimental environment and hyperparameters

This experiment was conducted on a Linux system based on the PyTorch deep learning framework and used NVIDIA GeForce RTX 3090 for GPU acceleration with 24G of memory. The programming software was VScode, and the programming language was Python. The specific experimental environment is shown in Table 1.

**Table 1:** Experimental environment.

environmental parameters	environment configuration
CPU	AMD EPYC 7K62 48-Core Processor
GPU	RTX 3090
GPU memory	24G
Operating System	Linux
CUDA	12.1
Python	3.10.14
Pytorch	2.1.0
Ultralytics	8.3.9

Additionally, the experimental hyperparameters used in this study are shown in Table 2.

**Table 2:** Experimental hyperparameters.

parameters	setpoint
epochs	300
Image size	640×640
Batchsize	8
Workers	4
optimizer	SGD
Initial learning rate	0.01
Final learning rate factor	0.01
Momentum	0.937
Weight decay	0.0005

### Evaluation metrics

To evaluate the model’s performance, the evaluation metrics used in the experimental section of this paper are Mean Average Precision (mAP), Giga Floating-point Operations Per Second (GFLOPS), and the number of parameters (Params). Among these, mAP50 represents the average precision of the model when the IOU threshold is set to 0.5. Specifically, mAP50 calculates the average of the average precision (AP) across all categories, where the average precision for each individual category is calculated at an IOU threshold of 0.5, serving as a specific evaluation criterion. The formula is as follows:

$$\text{mAP}=\frac{1}{n}\sum_{i=1}^{n}AP_{i}$$ (8)

Where *n* is the number of categories and *AP*_*i*_ is the average precision of the i-th category.

mAP50-95 is calculated using an IOU threshold ranging from 0.5 to 0.95, so it is often considered a more accurate reflection of the model’s performance at different stacking levels. As such, mAP50-95 is a stricter metric than mAP50. GFLOPS is typically used to measure the computational load of a model.

FPS (Frames Per Second) refers to the number of images that the model can process per second, which is an important indicator to measure the real-time performance of the model. The higher the FPS, the faster the model can process single-frame images, making it better suited for real-time detection requirements. All FPS data presented in Table 5 were obtained under the same experimental environment specified in Table 1.

## Experimental results and analysis

### Position experiment

First, we conducted a Position experiment on the C3k2-DMS-EdgeNet module, applying C3k2-DMS-EdgeNet separately to the backbone and neck, and finally using the C3k2-DMS-EdgeNet module in both the backbone and neck. The experimental results are shown in Table 3.

**Table 3:** Results of the position experiment.

Backbone	Neck	mAP50/%	mAP50-95/%	GFLOPS	Params/M
✓		75.5	58.4	6.4	2.56
	✓	74.8	57.5	6.7	2.65
✓	✓	76.1	58.7	6.8	2.63

To investigate the optimal placement of the C3k2-DMS-EdgeNet module, experiments were conducted by deploying it separately in the Backbone, Neck, and simultaneously in both. The results are shown in Table 3.

When the module was used only in the Neck, mAP50 and mAP50-95 decreased by 0.7% and 0.9%, respectively, compared to when it was used only in the Backbone, while the number of parameters and GFLOPS. This phenomenon may be due to the reduction in resolution when feature maps are passed into the Neck, causing the edge extraction function to fail.

When the module is used simultaneously in both the Backbone and Neck, mAP50 and mAP50-95 increase by 0.6% and 0.5%, respectively. The speculated reason is that the enhanced edge features in the Backbone are further strengthened in the Neck, but this also leads to an increase in computational complexity.

In summary, in the design of DMS-YOLO, considering the trade-off between computational cost and performance gains, we chose to use the module only in the Backbone, ensuring good detection performance while keeping the computation lighter.

### Ablation experiment

We conducted a total of six ablation experiments. First, YOLOv11n was trained and used as the baseline. Then, experiments were conducted by adding C3k2-DMS-EdgeNet, DySAN, and P2 improvements to this baseline. Finally, permutations and combinations were used for training. The results are shown in Table 4, where “✓” indicates that this method was used.

**Table 4:** Results of ablation experiments.

C3k2-DMS-EdgeNet	DySAN	P2	mAP50/%	mAP50-95/%	GFLOPS	Params/M
			73.3	57.6	6.3	2.58
✓			75.5	58.4	6.4	2.56
	✓		77.8	59.4	17.8	3.78
		✓	73.9	56.4	7.1	2.8
✓	✓		78.7	59.6	17.9	3.77
✓		✓	75.9	58.4	6.9	2.76
✓	✓	✓	80.3	60.5	17.9	3.77

As shown in the experimental results in Table 4, the improved C3k2 using DMS-EdgeNet not only slightly reduces the number of parameters but also increases mAP50 and mAP50-95 by 2.2% and 0.8%, respectively, indicating that the design of this module effectively enhances the ability to extract small object features while reducing the number of parameters. Introducing the DySAN module into Neck and performing multi-level jump connections increases the number of parameters by 1.2M, but also led to increases of 4.5% and 2.3% in mAP50 and mAP50-95, respectively, validating the effectiveness of the DySAN module in fusing deep and shallow feature information. Compared to the baseline model, The increase in parameters and computational complexity when introducing DySAN into the Neck may be due to the bidirectional enhancement of multi-level skip connections, but this also resulted in significant improvements in mAP50 and mAP50-95. The fifth and final set of ablation experiments showed that C3k2-DMS-EdgeNet and DySAN exhibit a strong cascading effect. The final improved DMS-YOLO achieves a substantial increase in average precision compared to the baseline model YOLOv11n, despite a 46% increase in the number of parameters.

### Comparative experiment

DMS-YOLO, YOLOv11s, m, and YOLOv12n, s were trained on the same dataset and in the same experimental environment. The experimental results are shown in Table 5.

As shown by the comparative experimental data in Table 5, compared to versions such as YOLOv11s and YOLOv11m, which have larger parameter counts and computational requirements, DMS-YOLO not only has fewer parameters and lower computational requirements but also demonstrates superior performance in terms of mAP50 and mAP50-95. Compared to the newer version YOLOv12s, which has a larger parameter count and computational requirements, DMS-YOLO also exhibits significant advantages.

**Table 5:** Results of comparative experimental.

Methods	mAP50/%	mAP50-95/%	GFLOPS	Params/M	FPS
YOLOv11n	73.3	57.6	6.3	2.58	666.7
YOLOv11s	76.5	59.8	21.3	9.4	394.8
YOLOv11m	79.2	61.5	67.7	20	216.2
YOLOv12n	70.8	56	5.8	2.5	625
YOLOv12s	78.9	60.3	19.3	9.07	381.6
DMS-YOLO(ours)	80.3	60.5	17.9	3.77	383.8

In terms of FPS performance, DMS-YOLO achieves 383.8 frames per second. Although it is lower than the lightweight models YOLOv11n and YOLOv12n, which achieve 666.7 FPS and 625 FPS respectively, DMS-YOLO still maintains high inference speed while significantly improving detection accuracy. Notably, compared with models with similar accuracy levels, DMS-YOLO shows remarkable speed advantages: compared with YOLOv11s (394.8 FPS) and YOLOv12s (381.6 FPS), DMS-YOLO achieves higher mAP values (80.3% mAP50 and 60.5% mAP50-95) while maintaining competitive inference speed (383.8 FPS). This indicates that DMS-YOLO has achieved an excellent balance between accuracy and real-time performance.

To further validate the effectiveness and robustness of DMS-YOLO, this paper selects other improved methods for comparison based on the second dataset, VisDrone-DET2019. Table 6 shows the experimental results of different algorithms under the same experimental environment.

**Table 6:** Comparison of detection results of different algorithms on the dataset VisDrone-DET2019.

Methods	mAP50/%	mAP50-95/%	GFLOPS	Params/M
YOLOv11n	28.3	16.1	6.3	2.58
YOLOv8s	31.8	17.6	28.6	11.1
YOLOv10s	32.1	18.5	21.6	8
YOLOv12n	27.6	15.9	5.8	2.5
SSD	23.6	12.9	24.5	87.9
Faster-RCNN	29.7	16.3	41.2	207.1
IM-YOLOv8 [28]	29.8	17.2	12.5	1.9
TPH-YOLO [29]	32.4	19.1	51.5	138.1
Swin Transformer [30]	30.6	17.4	44.5	34.2
DETR [31]	30.1	16.9	187.1	40.0
RT-DETR-R18 [32]	32.3	18.4	60.0	20.2
VRF-DETR [33]	34.9	20.1	44.3	13.5
UAV-DETR-EV2 [34]	34.5	19.8	43.0	13.0
DMS-YOLO(ours)	33.4	19.2	17.9	3.77

The data in Table 6 compares DMS-YOLO with representative detectors from previous related studies on the VisDrone-DET2019 dataset. Overall, DMS-YOLO achieves competitive detection accuracy (33.4% mAP50 and 19.2% mAP50-95) while maintaining relatively low computational cost (17.9 GFLOPS) and parameter count (3.77M). In particular, compared with representative one-stage detectors (e.g., SSD and Swin Transformer), a classic two-stage detector (Faster-RCNN), and improved YOLO variants (e.g., IM-YOLOv8 and TPH-YOLO), DMS-YOLO provides a more lightweight solution while preserving strong accuracy.

As a classic paradigm for end-to-end object detection, DETR exhibits significantly higher parameter counts and computational demands than DMS-YOLO, presenting limitations in lightweight adaptation. Its lightweight variant, RT-DETR-R18, achieves parameter and computational reduction while maintaining detection accuracy through optimizations to the backbone network and inference pipeline. Nevertheless, DMS-YOLO outperforms both DETR and its lightweight variant RT-DETR-R18 in terms of average precision. Variants like VRF-DETR and UAV-DETR, tailored for drone aerial photography scenarios, further optimize small object detection accuracy while reducing parameters and computational complexity. Although VRF-DETR and UAV-DETR slightly outperform DMS-YOLO on mAP50 and mAP50-95, their parameter counts and GFLOPS are approximately 3.5 times and 2.4 times those of DMS-YOLO, respectively. These comparisons highlight that DMS-YOLO achieves a favorable accuracy–efficiency trade-off.

### Small object performance evaluation

It is important to note that while the overall mAP50 metric evaluates detection performance across all object sizes, it does not specifically reflect the model’s capability on small objects alone. To address this, we introduce the mAP50-small metric to specifically evaluate the model’s performance on small object detection. This metric is calculated using the same approach as mAP50 described in the evaluation metrics section, where the IOU threshold is set to 0.5, but exclusively evaluates objects whose area ratio is less than 0.05 (i.e., objects occupying less than 5% of the total image area).

During evaluation on the test set of the Aerial Traffic Images dataset, we first filter both ground truth annotations and model predictions to retain only bounding boxes satisfying the small object criterion:

$$\text{area ratio}=\frac{w\times h}{\text{image width}\times \text{image height}}<0.05$$ (9)

where *w* and *h* represent the width and height of the bounding box. Subsequently, we calculate mAP50-small following the same computation procedure as mAP50 described in the evaluation metrics section, as follows:

$$\text{mAP50-small}=\frac{1}{n}\sum_{i=1}^{n}{AP}_{i}^{small}$$ (10)

where *n* is the number of object classes, and ${AP}_{i}^{small}$ denotes the Average Precision calculated on the filtered small objects of class *i* at IoU=0.5.

**Fig 9:**
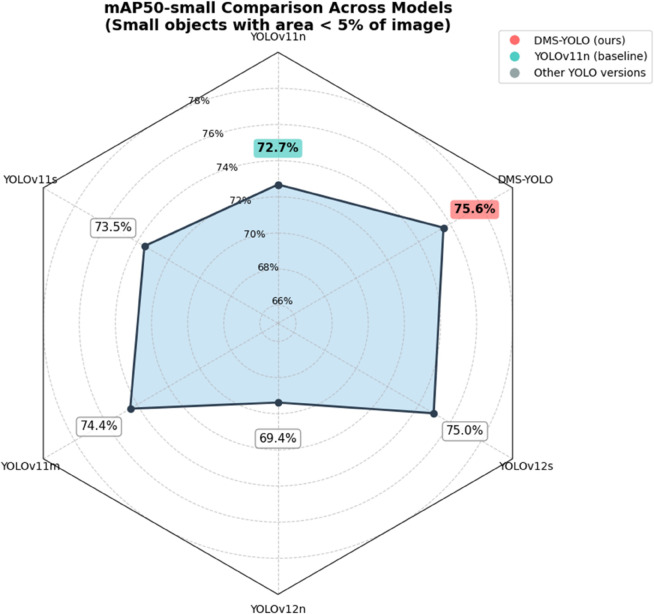
Comparison of mAP50-small across different YOLO versions.

The results are illustrated in Fig 9. DMS-YOLO achieves 75.6% mAP50-small on the Aerial Traffic Images test set, representing a 2.9% improvement over the baseline YOLOv11n (72.7%). Among all compared models, DMS-YOLO achieves the highest mAP50-small score. Compared to other models with larger parameter counts such as YOLOv11s (73.5%) and YOLOv11m (74.4%), DMS-YOLO demonstrates superior performance in small object detection while maintaining lower computational complexity.

The significant improvement in mAP50-small directly validates the effectiveness of DMS-YOLO’s small object-oriented design. These results conclusively demonstrate that the improvements in overall detection performance indeed stem from enhanced small object detection capability.

### Precision-recall curve analysis

**Fig 10:**
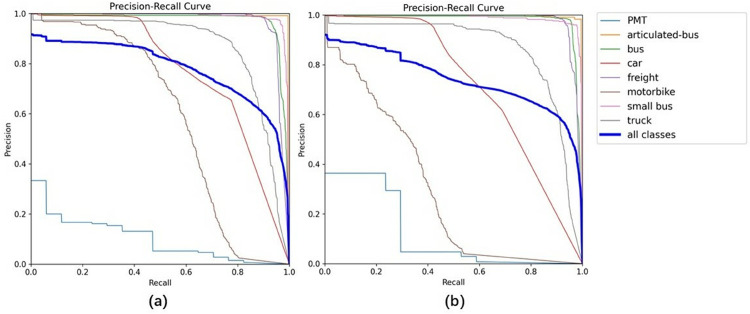
Precision-Recall curve comparison. (a): DMS-YOLO (b): YOLOv11n.

To further investigate the source of mAP improvement, this paper presents the Precision-Recall curves of DMS-YOLO and YOLOv11n on the Aerial Traffic Images dataset, as shown in Fig 10. The P-R curve is a widely used metric that simultaneously reflects both the precision and recall characteristics of a detection model across different confidence thresholds, with the area under the curve corresponding to the Average Precision (AP) value, the larger the area enclosed by the curve and the coordinate axes, the better the model’s detection performance for that category.

As illustrated in Fig 10, the overall performance curve of DMS-YOLO (represented by the blue bold line for all classes) demonstrates a fuller envelope, indicating superior detection performance compared to YOLOv11n. At a recall level of 0.4, DMS-YOLO maintains a precision exceeding 0.80, whereas YOLOv11n achieves approximately 0.80. As the recall increases to 0.6, the performance gap becomes more pronounced: DMS-YOLO sustains a precision of 0.80, while YOLOv11n declines to only 0.75.

The curve also reveals that DMS-YOLO extends to higher recall regions while maintaining competitive precision levels, demonstrating its capability to reduce missed detections. This characteristic is particularly important for small target detection in aerial imagery, where targets with blurred edges or low pixel density are prone to being overlooked.

Examining the individual category curves provides additional insights into the model’s performance across different object types.For challenging small object categories, particularly motorbike, DMS-YOLO exhibits notably smoother trajectory curves with precision values significantly higher than those of YOLOv11n, validating the effectiveness of the proposed improvements in addressing small target detection challenges. For larger object categories such as cars and buses, both models maintain stable high precision, with DMS-YOLO demonstrating marginal but consistent improvements across all recall levels.

For the PMT category (cyan line), which represents the most underrepresented class in the dataset with only 67 instances compared to over 64,000 car instances, a trade-off is observed: while DMS-YOLO shows slightly lower precision in the low recall region (0-0.3), its curve extends to significantly higher recall levels compared to YOLOv11n. This demonstrates that DMS-YOLO can successfully detect a larger proportion of minority class instances, indicating better handling of severe class imbalance.

In summary, the mAP improvement is driven by balanced enhancements in both precision and recall across all object categories.

### Visual analytics

**Fig 11:**
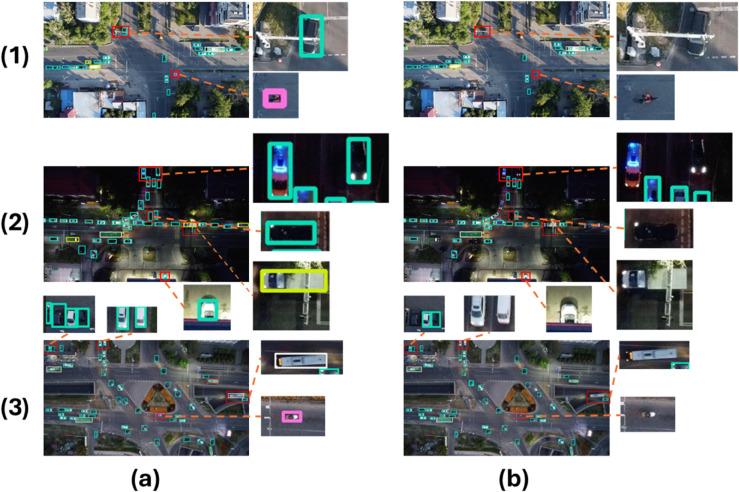
Detection results in different scenarios. (a): DMS-YOLO detection results. (b): YOLOv11n detection results.

To demonstrate the effectiveness of the model improvement, this paper compares the detection results of the baseline model YOLOv11n and DMS-YOLO on the Aerial Traffic Images test set. The results are shown in Fig 11. In the first image, the baseline model YOLOv11n has missed detection issues, with targets located at the edges being missed due to occlusion, and motorcycles located at the center being missed due to their small scale. In the second image, YOLOv11n also fails to detect the truck due to the dark environment and insufficient lighting, and the car at the bottom of the image is also missed due to occlusion. In the third image, YOLOv11n completely misses both the small bus and the motorcycle, and some vehicles are also missed due to their black color and the dim lighting at the tunnel entrance. In summary, the algorithm designed in this paper maintains good recognition accuracy in scenarios with large target scale variations and blurred target edges, validating the effectiveness of the DMS-EdgeNet and DySAN designs.

### Heat map analysis

**Fig 12:**
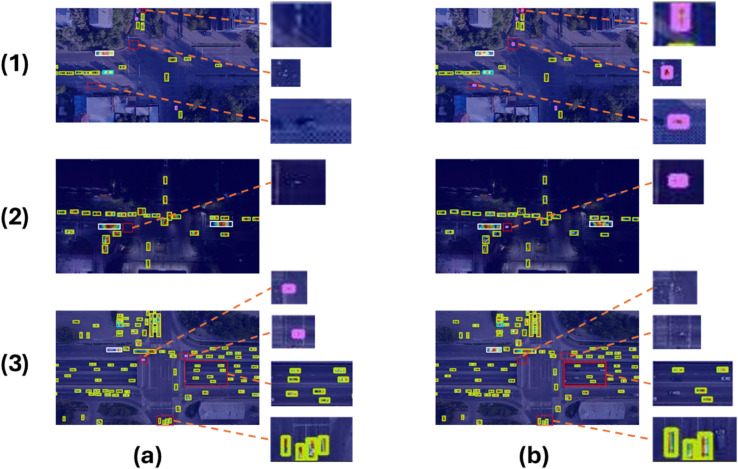
Comparison of heat maps. (a): DMS-YOLO detection results. (b): YOLOv11n detection results.

To further demonstrate the advantages of DMS-YOLO in small object detection tasks, a heatmap analysis was performed on the images. As shown in Fig 12, in the first image, YOLOv11n incorrectly detected the center line of the road and pedestrians as a motorcycle. In the second image, YOLOv11n incorrectly detected a streetlight as a motorcycle. In the third image, YOLOv11n failed to detect the motorcycle and also missed several vehicles on the right side of the image that were similar in color to the road. In contrast, DMS-YOLO detected more small objects and demonstrated excellent capability in extracting detailed information about the objects, thereby reducing the probability of false positives and false negatives. This further validates the superiority of DMS-YOLO in small object detection tasks.

## Discussion

The comparison across different YOLO versions and sizes shows that DMS-YOLO improves detection accuracy over the lightweight baseline YOLOv11n. Compared with larger YOLO variants (e.g., YOLOv11s/m and YOLOv12s), DMS-YOLO achieves competitive accuracy with fewer parameters and lower computational cost, indicating a favorable accuracy–efficiency trade-off.

In addition to comparisons within the YOLO series, this paper further compares DMS-YOLO with previous related studies, including representative CNN-based detectors and recent transformer-based variants. While some UAV-oriented DETR variants achieve slightly higher mAP, they require substantially higher computational resources and parameter counts. Therefore, the advantages of DMS-YOLO are best reflected by its favorable accuracy–efficiency trade-off, which is important for real-time UAV applications with limited compute budgets.

The small-object performance evaluation section provides direct evidence that the overall mAP improvement is largely attributed to improved small-object detection performance on the Aerial Traffic Images dataset. Specifically, DMS-YOLO achieves a higher mAP50-small than the YOLOv11n baseline under the same small-object criterion, indicating that the proposed design improves feature representation for small targets. The precision–recall curve analysis further shows that DMS-YOLO maintains higher precision at the same recall and extends to higher recall regions, suggesting fewer missed detections; this trend is consistent with the qualitative visualizations and heat map analysis, where DMS-YOLO detects more small objects and reduces false positives/false negatives in low-light scenes.

Despite these improvements, the results also reveal several limitations that are closely related to the characteristics of the aerial datasets used in this study. First, the Aerial Traffic Images dataset exhibits severe class imbalance: the public-transport category (PMT) contains only 67 instances, whereas the car category contains more than 64,000 instances. This large gap can bias training toward majority classes and makes it difficult to learn robust representations for minority classes, leading to noticeably lower detection performance for PMT. Second, although both datasets include many low-light scenes, they lack explicitly labeled adverse-weather subsets (e.g., heavy fog and rain), which limits a systematic evaluation of robustness under challenging weather conditions. These limitations provide clear directions for future work, including better handling of long-tailed class distributions and expanding evaluation with weather-annotated UAV datasets.

## Conclusion

This paper proposes an improved structure based on YOLOv11n, DMS-YOLO, to address the issue that the same detection target appears in different sizes in different images due to variations in height, angle, and perspective when capturing images from a drone, as well as the inaccurate localization of edge-blurred targets in UAV aerial imagery. To address these issues, this paper designs DMS-EdgeNet to replace the Bottleneck module of C3k2 in the original network, aiming to strengthen multi-scale feature representation and enhance edge-aware cues for small objects, and selects the C2f structure for the first two C3k2 modules in the backbone network to reduce computational load while maintaining accuracy. Additionally, the DySAN module is designed to autonomously fuse deep and shallow features to finetune target boundaries and enhance boundary features, and multi-level skip connections are employed to reduce information decay during single-fusion processing. Finally, a P2 small object detection layer is introduced to further enhance the model’s feature extraction ability for small objects.

Compared with the baseline YOLOv11n, DMS-YOLO improves mAP50/mAP50-95 by 7.0%/2.9% on the Aerial Traffic Images dataset, and by 5.1%/3.1% on the VisDrone-DET2019 dataset.

To further explain the source of the mAP improvement, this paper introduces the mAP50-small metric to specifically evaluate small-object detection. DMS-YOLO achieves 75.6% mAP50-small on the Aerial Traffic Images test set, which is a 2.9% improvement over YOLOv11n (72.7%). These results indicate that the overall mAP gains are largely driven by improved detection accuracy for small objects in the dataset.

## References

[1] Chinnari P, Sinha S, Ishaa B, Bharill N, Patel OP. Semantic segmentation in aerial imagery: a novel approach for urban planning and development. In: 2024 IEEE 48th Annual Computers, Software, and Applications Conference (COMPSAC). 2024. p. 1618–23. 10.1109/compsac61105.2024.00254

[2] Yang K, Zhang S, Yang X, Wu N. Flood detection based on unmanned aerial vehicle system and deep learning. Complexity. 2022;2022(1). doi: 10.1155/2022/6155300

[3] Messenger R, Islam MZ, Whitlock M, Spong E, Morton N, Claggett L. Real-time traffic end-of-queue detection and tracking in UAV video. arXiv preprint 2023. https://arxiv.org/abs/2302.01923

[4] Wang J, Zhao C, Huo Z, Qiao Y, Sima H. High quality proposal feature generation for crowded pedestrian detection. Pattern Recognition. 2022;128:108605. doi: 10.1016/j.patcog.2022.108605

[5] Viola P, Jones M. Rapid object detection using a boosted cascade of simple features. In: Proceedings of the 2001 IEEE Computer Society Conference on Computer Vision and Pattern Recognition. CVPR 2001. p. I-511-I–518. 10.1109/cvpr.2001.990517

[6] Felzenszwalb PF, Girshick RB, McAllester D, Ramanan D. Object detection with discriminatively trained part-based models. IEEE Trans Pattern Anal Mach Intell. 2010;32(9):1627–45. doi: 10.1109/TPAMI.2009.167 20634557

[7] Dalal N, Triggs B. Histograms of oriented gradients for human detection. In: 2005 IEEE Computer Society Conference on Computer Vision and Pattern Recognition (CVPR’05). p. 886–93. 10.1109/cvpr.2005.177

[8] Khanam R, Hussain M. YOLOv11: an overview of the key architectural enhancements. 2024. https://arxiv.org/abs/2410.17725

[9] Liu W, Anguelov D, Erhan D, Szegedy C, Reed S, Fu CY, et al. SSD: Single Shot MultiBox Detector. In: Leibe B, Matas J, Sebe N, Welling M, editors. Computer Vision – ECCV 2016. Cham: Springer; 2016. p. 21–37.

[10] Ren S, He K, Girshick R, Sun J. Faster R-CNN: towards real-time object detection with region proposal networks. IEEE Trans Pattern Anal Mach Intell. 2017;39(6):1137–49. doi: 10.1109/TPAMI.2016.2577031 27295650

[11] Liu L, Li J. MCRS-YOLO: multi-aggregation cross-scale feature fusion object detector for remote sensing images. Remote Sensing. 2025;17(13):2204. doi: 10.3390/rs17132204

[12] Di X, Cui K, Wang R-F. Toward efficient UAV-based small object detection: a lightweight network with enhanced feature fusion. Remote Sensing. 2025;17(13):2235. doi: 10.3390/rs17132235

[13] Weng S, Wang H, Wang J, Xu C, Zhang E. YOLO-SRMX: a lightweight model for real-time object detection on unmanned aerial vehicles. Remote Sensing. 2025;17(13):2313. doi: 10.3390/rs17132313

[14] Lin TY, Maire M, Belongie S, Bourdev L, Girshick R, Hays J. Microsoft COCO: common objects in context. 2015. https://arxiv.org/abs/1405.0312

[15] Xia GS, Bai X, Ding J, Zhu Z, Belongie S, Luo J, et al.. DOTA: a large-scale dataset for object detection in aerial images; 2019. https://arxiv.org/abs/1711.10398 10.1109/TPAMI.2021.3117983 34613910

[16] Cao B, Yao H, Zhu P, Hu Q. Visible and clear: finding tiny objects in difference map. 2024. https://arxiv.org/abs/2405.11276

[17] Guo F, Wu J, Zhang Q. Dual-domain feature-guided task alignment for enhanced small object detection. In: ICASSP 2025 - 2025 IEEE International Conference on Acoustics, Speech and Signal Processing (ICASSP). 2025. p. 1–5.

[18] Guo Z, Shuai H, Liu G, Zhu Y, Wang W. Multi-level feature fusion pyramid network for object detection. Vis Comput. 2022;39(9):4267–77. doi: 10.1007/s00371-022-02589-w

[19] Liu Z, Gao G, Sun L, Fang Z. HRDNet: high-resolution detection network for small objects. In: 2021 IEEE International Conference on Multimedia and Expo (ICME). 2021. p. 1–6. 10.1109/icme51207.2021.9428241

[20] Li Y, Li Q, Pan J, Zhou Y, Zhu H, Wei H, et al. SOD-YOLO: small-object-detection algorithm based on improved YOLOv8 for UAV images. Remote Sensing. 2024;16(16):3057. doi: 10.3390/rs16163057

[21] Peng H, Xie H, Liu H, Guan X. LGFF-YOLO: small object detection method of UAV images based on efficient local–global feature fusion. J Real-Time Image Proc. 2024;21(5). doi: 10.1007/s11554-024-01550-5

[22] Zeng S, Yang W, Jiao Y, Geng L, Chen X. SCA-YOLO: a new small object detection model for UAV images. Vis Comput. 2023;40(3):1787–803. doi: 10.1007/s00371-023-02886-y

[23] Tong K, Wu Y. I-YOLO: a novel single-stage framework for small object detection. Vis Comput. 2024;40(12):8927–44. doi: 10.1007/s00371-024-03284-8

[24] Li X, Li X, Zhang L, Cheng G, Shi J, Lin Z, et al. Improving semantic segmentation via decoupled body and edge supervision. Lecture Notes in Computer Science. Springer; 2020. p. 435–52. 10.1007/978-3-030-58520-4_26

[25] Hu J, Shen L, Sun G. Squeeze-and-excitation networks. In: 2018 IEEE/CVF Conference on Computer Vision and Pattern Recognition. 2018. p. 7132–41. 10.1109/cvpr.2018.00745

[26] Wang Q, Yu C. AMFE-YOLO: a small object detection model for drone images. IET Image Processing. 2025;19(1):e70110.doi: 10.1049/ipr2.70110

[27] Du D, Zhu P, Wen L, Bian X, Lin H, Hu Q, et al. VisDrone-DET2019: the vision meets drone object detection in image challenge results. In: 2019 IEEE/CVF International Conference on Computer Vision Workshop (ICCVW); 2019. p. 213–26.

[28] Wu Q, Li X, Xu C, Zhu J. An improved YOLOv8n algorithm for small object detection in aerial images. In: 2024 9th International Conference on Signal and Image Processing (ICSIP). 2024.pP. 607–11. 10.1109/icsip61881.2024.10671469

[29] Zhu X, Lyu S, Wang X, Zhao Q. TPH-YOLOv5: improved YOLOv5 based on transformer prediction head for object detection on drone-captured scenarios. In: 2021 IEEE/CVF International Conference on Computer Vision Workshops (ICCVW). 2021. p. 2778–88.

[30] Liu Z, Lin Y, Cao Y, Hu H, Wei Y, Zhang Z, et al. Swin transformer: hierarchical vision transformer using shifted windows. In: 2021 IEEE/CVF International Conference on Computer Vision (ICCV). 2021; p. 9992–10002.

[31] Carion N, Massa F, Synnaeve G, Usunier N, Kirillov A, Zagoruyko S. End-to-end object detection with transformers. In: Computer Vision – ECCV 2020 : 16th European Conference, Glasgow, UK, August 23–28, 2020, Proceedings, Part I, 2020. p. 213–29. 10.1007/978-3-030-58452-8_13

[32] Zhao Y, Lv W, Xu S, Wei J, Wang G, Dang Q, et al. DETRs beat YOLOs on real-time object detection. In: 2024 IEEE/CVF Conference on Computer Vision and Pattern Recognition (CVPR). 2024. p. 16965–74. 10.1109/cvpr52733.2024.01605

[33] Liu W, Shi L, An G. An efficient aerial image detection with variable receptive fields. Remote Sensing. 2025;17(15):2672. doi: 10.3390/rs17152672

[34] Zhang H, Liu K, Gan Z, Zhu GN. UAV-DETR: efficient end-to-end object detection for unmanned aerial vehicle imagery. 2025. https://arxiv.org/abs/2501.01855

